# Effects of Benson Relaxation Technique and Music Therapy on the Anxiety of Primiparous Women Prior to Cesarean Section: A Randomized Controlled Trial

**DOI:** 10.1155/2022/9986587

**Published:** 2022-12-23

**Authors:** Sedigheh Nosrati Abarghoee, Abbas Mardani, Robabe Baha, Nasrin Fadaee Aghdam, Mahboobeh Khajeh, Fatemeh Eskandari, Mojtaba Vaismoradi

**Affiliations:** ^1^Member of the Department of Anesthesiology, Faculty of Paramedicine, Alborz University of Medical Sciences, Karaj, Iran; ^2^Nursing and Midwifery Care Research Center, Department of Medical Surgical Nursing, School of Nursing and Midwifery, Iran University of Medical Sciences, Tehran, Iran; ^3^Alborz University of Medical Sciences, Karaj, Iran; ^4^School of Nursing and Midwifery, Shahroud University of Medical Sciences, Shahroud, Iran; ^5^HammondCare. Mental Health/ Specialized Dementia Care Worker, Sydney, Australia; ^6^Faculty of Nursing and Health Sciences, Nord University, Bodo, Norway

## Abstract

**Background and Aims:**

Primiparous women experience high levels of anxiety before cesarean section. Therefore, this research aimed to investigate the effects of the Benson Relaxation Technique (BRT) and Music Therapy (MT) on the anxiety of primiparous women prior to cesarean section.

**Methods:**

A randomized controlled trial was carried out on 105 women scheduled for cesarean section. They were randomly assigned into three groups: BRT, MT, and control (*n* = 35 per group). The women in the BRT and MT groups performed exercises and listened to music, respectively, for 20 minutes prior to cesarean section. The State Anxiety Inventory was used to measure the women's anxiety in the groups before and after the intervention.

**Results:**

Within-group comparisons showed that the women in the BRT (*t* = 5.61, *p* < 0.001, effect size (Cohen's *d*) = 0.94) and MT (*t* = 3.83, *p* = 0.001, *d* = 0.64) groups had significantly lower anxiety after the interventions compared to before the interventions. Also, between-group comparisons revealed that anxiety after the intervention was significantly lower in the BRT and MT groups compared to the control group (*p* = 0.007).

**Conclusions:**

Although both of the BRT and MT helped with the reduction of anxiety among primiparous women before cesarean section, the BRT was shown more effective. These nonpharmacologic methods are safe and cost-effective and can improve well-being among women undergoing this invasive procedure. They can be used along with pharmacologic methods for reducing overreliance on medications.

## 1. Introduction

The rate of cesarean section as a common surgical procedure has increased across the globe in the last decades [1, 2]. A recent statistic from 150 countries shows that 18.6% of all childbirths happen by cesarean section, ranging from 1.4% to 56.4%. Also, the cesarean section in Iran has the prevalence rate of 47.9%, which is higher than in other countries [3].

Women undergoing cesarean section mostly have high levels of preoperative anxiety [4–6]. Accordingly, 63–86% of women undergoing cesarean section experience preoperative anxiety, that is higher than anxiety before general surgeries [7–9]. Parity can affect childbirth anxiety. The results of previous studies on childbirth anxiety are inconsistent [10, 11], but it has been shown that primiparous women experience more anxiety than multiparous women at childbirth [4, 12]. Distress and anxiety are associated with the selection of cesarean section over vaginal delivery as the childbirth method by women [13]. Anxiety before cesarean section can influence satisfaction with childbirth, delay recovery, and the maternal perception of pain [14]. Also, it can increase requests for analgesic medications and length of hospital stay [15, 16]. Moreover, the level of perceived anxiety before childbirth is significantly associated with postpartum hemorrhage [17], postpartum bruxism, and temporomandibular disorder [18–22]. Women's mental state can have adverse effects on the fetus leading to fetal asphyxia, abnormal fetal heart rate patterns, low Apgar scores, and increased mortality [23].

Pharmacological methods are commonly used to manage preoperative anxiety among women before cesarean section. Most antianxiety medications can pass via the placenta and cause negative consequences for the fetus [24]. Therefore, the use of nonpharmacological methods for reducing anxiety among women undergoing cesarean section has been emphasized [24–27]. For instance, the effect of relaxation techniques on the alleviation of preoperative anxiety has been reported [28–30].

The Benson relaxation technique (BRT) is a common relaxation technique and has been recognized as one of the most appropriate and cost-effective methods for reducing health problems [31–33]. The accomplishment of relaxation does not require any special tool or physician's prescription and it can be used in various situations [34, 35]. The BRT, aside from its numerous advantages and the simplicity of its implementation, has no side effects on patients [36, 37]. The effect of the BRT on patient's anxiety has been investigated in patients with different health problems. For instance, BRT can decrease anxiety symptoms in patients undergoing hemodialysis [38], those admitted to the emergency department [39], with coronary artery disease [40], surgery candidates [28], and undergoing mastectomy [41].

Music therapy (MT) is another nonpharmacologic method for relieving patients' anxiety. The use of MT as a treatment method has a long history [42, 43]. Its effectiveness to reduce anxiety in patients with different health problems such as oncologic surgery [44], chronic obstructive pulmonary disease [45], and gynecological surgery [46] have been reported.

### 1.1. Objectives and Hypotheses

Preoperative anxiety and the use of medications for relieving it has negative consequences for the mother and the fetus. There is a need for safe methods that can reduce maternal anxiety and improve their wellbeing in women undergoing cesarean section. Therefore, this study aimed to investigate the effects of the BRT and MT on the anxiety of primiparous women prior to cesarean section. The research hypotheses were:

1. The BRT and MT relieve anxiety among primiparous women prior to cesarean section compared to the control group
2. The anxiety level among primiparous women receiving the BRT is similar to primiparous women receiving MT

## 2. Methods

### 2.1. Study Design and Setting

A parallel, three-armed, randomized controlled trial was performed. Pregnant women scheduled for cesarean section in four public hospitals in an urban area of Iran were the study participants. The study was carried out from September 2021 to December 2021. This article has been reported using the CONsolidated Standards of Reporting Trials (CONSORTs) (Supplementary file 1).

### 2.2. Sampling and Recruitment

Eligibility criteria for the selection of the participants were: primiparous; full consciousness; abilities to read and write in Farsi; no history of surgical procedures, mental illness, anxiety disorder, and hearing impairment; no experience of the use of relaxation methods in the past or taking herbal or medical sedatives; an anxiety score above 31 on the State Anxiety Inventory (SAI) [47] indicating a moderate to severe anxiety level. Changes in the women's and their fetuses' hemodynamic conditions, the need for additional medical care before cesarean section, and unwillingness to cooperate during the interventions led to their exclusion.

**Figure 1:**
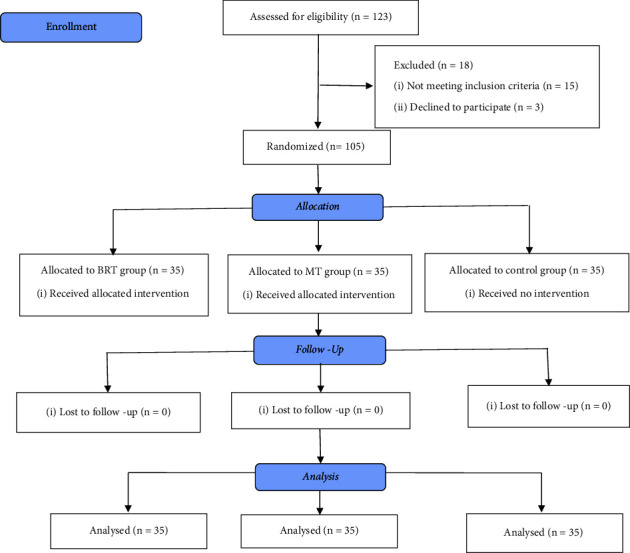
The process of the study is according to the CONSORT flow diagram (2010).

Based on the results of previous studies [36, 41], considering *α* = 0.05 and *β* = 80%, the sample size was estimated at 35 people in each group. After obtaining permissions from the university and hospital officials, the main researcher referred to the selected hospitals. A consecutive sampling method was used for the selection of women scheduled for cesarean section. Eligible participants were approached and sufficient explanations about the research aim and process were presented to them. Willing participants were included in the study and were randomly assigned to three groups as the BRT, MT, and control using the shuffling method via cards placed in opaque envelopes [48]. Code “A” was named as the BRT, code “B” as the MT, and code “C” as the control. The letters A, B, and C were written on cards and were placed in opaque envelopes. Each of the enrolled participants was requested to pick one of the envelopes to be assigned to the groups (Figure 1).

### 2.3. Data Collection

The demographic data questionnaire and the State Anxiety Inventory (SAI) were used.

#### 2.3.1. Demographic Data Questionnaire

It included questions about the participants' age, marriage duration, level of education, job status, economic status, and living place. It was completed by the participants before the interventions in the women's surgical ward.

#### 2.3.2. State Anxiety Inventory (SAI)

It contains 20 items that are scored using a four-point Likert scale ranging from 1 “not at all” to 4 “very much”. The range of scores is from 20 to 80, which is categorized from mild anxiety (score 20–31) to very severe anxiety (score above 76) [47]. For reliability, Cronbach's alpha coefficient and the test-retest reliability coefficient are reported 0.90 and 0.62, respectively [49]. The reliability of the Farsi version of SAI has been confirmed with a Cronbach's alpha coefficient of 0.90. The correlation between the observed and the real score is 0.97 [50]. The participants' anxiety was measured before and immediately after the interventions in the women's surgical ward.

### 2.4. Intervention

The interventions were performed two hours before the cesarean section. The participants in the BRT group were individually trained by the main researcher about how to perform this technique. To ensure the correct implementation of the technique, the participants were asked to explain it back to the researcher and modifications were made. In addition, the BRT was audio-recorded and was given to the participants to be played by an MP3 player in order to ensure of its correct implementation.

The BRT included four main principles. (i) Being placed in a quiet room in a perfectly comfortable position; (ii) taking a position and holding it for at least 20 minutes; (iii) having a mental focus on a subject or object through the repetition of a word or sound, or a pleasant feeling; (iv) having a positive attitude and mind free from thoughts, worries, and disturbances [33, 51]. The participants were placed in comfortable positions and were asked to close their eyes and focus on their breathing, to remove disturbing thoughts from their minds as much as possible. They were asked to choose a word that always reminded them of peace such as God, love, sea, and rainbow, and to start breathing deeply and regularly, inhale through the nose, exhale through the mouth, and repeat the selected sedative word. At the same time, they relaxed their muscles from the tips of their toes and continued them toward the upper body and head muscles until all muscles became fully relaxed. They were also asked not to think about the effectiveness of this method and to continue this exercise for at least twenty minutes. They were asked to open their eyes and stand still for a while to achieve the desired relaxation.

The MT group listened to nonverbal music, which was played for them for 20 minutes through an MP3 player. The song “Weightless,” by Macaroni Union, was selected and played as the nonverbal music for the MT group. Melodies in this song have no repeated pattern and steadily slow from sixty beats to fifty beats per minute over its 8 minutes duration [52].

The BRT and MT interventions were performed in the women's ward and in a private room. The “please do not enter” sign was hung on the participant's room door and their companions were asked to leave the room during the interventions. The participants in the control group only received routine nursing care. The control group received routine care that focused on a detailed explanation of the surgical procedure and recovery after cesarean section.

### 2.5. Ethical Considerations

The ethical approval was granted by the ethics committee of Alborz University of Medical Sciences (decree code: IR.ABZUMS.REC.1397.163). Also, the research protocol was registered at the Iranian Registry of Clinical Trials (IRCT) (code: IRCT20190408043199N1). Permissions to enter the hospitals were obtained from the relevant authorities. The participants were informed about their right to leave the study at any time without any consequence on their care. They were also assured of data confidentiality and their anonymity. The written consent form was signed by the participants before the study. Blinding of the researcher and the participants was impossible due to the nature of the interventions. However, the statistical specialist was blind to the group assignments to avoid bias during the data analysis.

### 2.6. Data Analysis

Descriptive statistics including mean, standard deviation, frequency, and percentage were used for summarizing the data. The Kolmogorov–Smirnov test was used to assess the normality of the data. A comparison of demographic characteristics between the study groups was conducted using the one-way ANOVA test and Chi-squared test. The One-way ANOVA test and paired samples *t*-test were used to compare between and within the groups' anxiety scores. To calculate the effect size as Cohen's *d*, the difference between the groups' means was divided into the pooled standard deviation. The data were analyzed using the SPSS v.25 and the significance level was set at *p* < 0.05.

## 3. Results

The eligibility of 123 women were assessed of which 15 women did not meet the criteria, and three women declined to participate. Therefore, 105 women were randomly assigned to the BRT (*n* = 35), MT (*n* = 35), and control (*n* = 35) groups. During the interventions, no sample dropping out was observed and data collected from all participants were used for the data analysis.

### 3.1. Demographic Characteristics of the Participants

The mean (SD) age of the participants in the BRT, MT, and control groups were 26.94 years (6.50 years), 29.97 (7.66), and 29.34 (6.68), respectively. The range of marriage duration was between 1 and 10 years with a mean (SD) of 5.25 y (2.66 y) in the BRT group, 4.94 (2.58) in the MT group, and 5.11 (2.69) in the control group. The highest frequency of educational level in the groups was for those under diploma. Other demographic characteristics have been listed in Table 1. No significant differences in the demographic characteristics between the groups were reported (*p* > 0.05).

### 3.2. Anxiety

The mean scores and SD of anxiety before and after the interventions in the groups have been presented in Table 2.

Within-group comparisons showed that the participants in both the BRT (*t* = 5.61, *p* < 0.001, effect size (Cohen's *d*) = 0.94), and MT (*t* = 3.83, *p* = 0.001, *d* = 0.64) groups had statistically significant lower anxiety after the interventions compared to before the interventions. There was no statistically significant difference in the anxiety level before and after the interventions in the control group (*t* = 1.21, *p* = 0.23, Cohen's *d* = 0.20).

**Figure 2:**
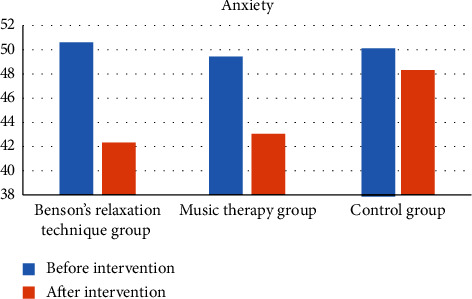
Comparison of anxiety between the study groups.

The between-group comparison revealed no statistically significant difference in anxiety between the BRT, MT, and control groups before the interventions (*F* = 0.17, *p* = 0.83). However, a statistically significant difference was observed in anxiety between the groups after the interventions (*F* = 5.16, *p* = 0.007). Multiple comparisons of Bonferroni showed a statistically significant difference between the BRT group (mean difference (MD) = −6.00, *p* = 0.01) and the MT group (MD = −5.25, *p* = 0.03) compared to the control group (Table 2). There was no evidence of complications resulting from the interventions or dissatisfaction with them in the intervention groups.  Figure 2 illustrates changes in anxiety before and after the interventions in the groups.

## 4. Discussion

This study aimed to investigate and compare the effects of the BRT and MT on anxiety among primiparous women before cesarean section. The results showed that the women's anxiety significantly decreased in the BRT and the MT groups compared to the control group.

The participants in the BRT group reported significantly lower anxiety after the intervention. Consistent with our findings, two recent studies from Iran [23] and Indonesia [53] reported that BRT reduced women's anxiety during the precesarean section period. Also, the effectiveness of the BRT on the reduction of anxiety before cataract surgery [54], gastrointestinal surgery [55], open-heart surgery [56], and angiography [57] were reported.

Anxiety, as an unpleasant mental feeling, is a natural response to potential dangers that stimulate the autonomic nervous system and trigger hormonal changes [4, 58]. Modulating neurohormonal responses in the hypothalamic, pituitary, and adrenal axes and increasing serotonin levels can regulate the secretion of cortisol and cause relaxation. Also, serotonin through facilitating the secretion of gamma-aminobutyric acid (GABA) leads to calming and modulating behavioral responses [59]. Moreover, reducing the activity of the sympathetic system and the secretion of endorphins play important roles in calming the person [60]. The BRT regulates the hypothalamus, inhibits sympathetic activity, reduces adrenaline, and increases endorphins [61] and serotonin levels [62]. Therefore, through these mechanisms anxiety can be alleviated [56].

According to our study findings, after the intervention, the participants in the MT group reported significantly lower anxiety. Similarly, the study by Kushnir et al. in Israel showed that listening to favorite music immediately prior to cesarean section decreased negative emotions including anxiety [25]. In addition, a study on Chinese women indicated that a preoperative music intervention reduced anxiety before cesarean section [63]. Another study revealed that MT not only had an effect on precesarean section anxiety but also had a lasting effect, and patients benefited from its antianxiety effects even after surgery [64]. Music can increase the stress threshold, eliminate negative emotions, regulate internal processes, create relaxed and calm states, and increase immunity, as well as the integration of psychosocial, physiological, and emotional states [65]. In addition, MT can provide direct physiological, psychological, and social-emotional benefits for patients [66]. This antianxiety effect of MT can be due to stimulating the brain and the release of hormones such as serotonin and endorphins [67].

In the present study, both the BRT and MT were effective in reducing the women's anxiety, but the BRT had more effect. The researchers found only one study comparing the effects of BRT and MT on anxiety. In an Iranian study [68], MT had a greater effect compared to the BRT on decreasing patients' anxiety levels before cardiac catheterization. This inconsistency may be due to difference in the method of MT. For instance, patients in the MT group had the right to choose music from four types of music [68] and also the nature of anxiety in the studies' partcipants was different. A previous study revealed that nature sounds and relaxation exercises were similarly effective in reducing preoperative anxiety [30]. However, further studies are needed to examine differences between BRT and MT in reducing preoperative anxiety.

In addition to the benefits of reducing precesarean anxiety using the BRT and MT, they also can have a notable effect on maternal and fetal outcomes. By stimulating the secretion of endorphins, these interventions can relieve pain, and by activating reward and pleasure centers in the brain, they can promote maternal-neonate contact, as well as promote the beginning of the breastfeeding process [69–71].

As the limitation of this study, it was impossible to provide a very quiet environment for conducting the intervention, which might have impacted anxiety in the participants. Moreover, anxiety was measured subjectively using a self-report questionnaire. Therefore, a more objective method for anxiety measurement should be used to reflect the effects of the interventions with more reliability.

### 4.1. Implication for Practice

One of the important roles of nurses during patient care is to identify patients' anxiety and apply the most effective method to improve their wellbeing [72, 73]. Also, nurses have the responsibility to consider the safety of the anxiety relieving method in patient care. Primiparous women often experience high anxiety levels in the precesarean period. Based on our study findings, both BRT and MT were effective in reducing precesarean anxiety, but BRT was more effective. The BRT and MT are cost-efficient, have no side effects, and are noninvasive. Therefore, it is suggested to use these interventions to decrease anxiety among pregnant women prior to cesarean section.

## 5. Conclusion

The BRT and MT can decrease anxiety in primiparous women before cesarean section. As safe and nonpharmacological methods, they can be used to reduce anxiety levels among them. Investigations to determine the persistence of the effects of these methods on anxiety during and after cesarean section is suggested. Future studies are suggested to investigate the combined effects of the BRT and MT on anxiety in primiparous women before cesarean section.

## Figures and Tables

**Table 1:** The demographic characteristics of the participants in the groups.

Variables	Groups *N* (%)			*p* value
	BRT	MT	Control	
Age; mean (SD), year	26.94 (6.50)	29.97 (7.66)	29.34 (6.68)	0.16^a^
Marriage duration; mean (SD), year	5.25 (2.66)	4.94 (2.58)	5.11 (2.69)	0.88^a^
Education level				0.27^b^
Under diploma	19 (54.3)	20 (57.1)	22 (62.9)	
Diploma	4 (11.4)	8 (22.9)	8 (22.9)	
Academic	12 (34.3)	7 (20)	5 (14.3)	
Job status				0.08^b^
Housewife	26 (74.3)	19 (54.3)	27 (77.1)	
Employed	9 (25.7)	16 (45.7)	8 (22.9)	
Economic status (self-report)				0.99^b^
Weak	12 (34.3)	2 (5.7)	9 (25.7)	
Fair	9 (25.7)	12 (34.3)	17 (48.6)	
Good	14 (40)	20 (57.1)	9 (25.7)	
Living place				0.33^b^
City	34 (97.1)	31 (88.6)	31 (88.6)	
Village	1 (2.9)	4 (11.4)	4 (11.4)	

BRT: Benson's relaxation technique; MT: Music therapy^a^One-way ANOVA test^b^Chi-squared test.

**Table 2:** Mean scores of anxiety before and after the interventions in the groups.

Variable	Time	Groups			*F* value	*p* value^a^
		BRT	MT	Control		
		Mean (SD)	Mean (SD)	Mean (SD)		
Anxiety	Before the intervention	50.60 (1.30)	49.42 (1.61)	50.25 (1.36)	0.17	0.83
	After the intervention	42.31 (1.33)	43.05 (1.19)	48.31 (1.73)	5.16	0.007

Intragroup comparison	*t* value	5.61	3.83	1.21		
	*p* value^b^	<0.001	0.001	0.23		
	Effect size (Cohen's *d*)	0.94	0.64	0.20		

BRT: Benson's relaxation technique; MT: Music therapy^a^One-way ANOVA test;^b^ Paired samples *t*-test.

## Data Availability

The complementary data used to support this study are available from the corresponding author upon reasonable request.

## Abbreviations

BRT: Benson's relaxation technique
MT: Music therapy
SAI: State anxiety inventory.

## Supplementary Materials

Supplementary Materials The CONSORT checklist is presented in the supplementary file.
